# FI-Lab predicts all-cause mortality, but not successful rehabilitation, among patients aged 65 and older after hip or pelvic fracture

**DOI:** 10.3389/fmed.2025.1627026

**Published:** 2025-07-16

**Authors:** Evgeniya Zikrin, Abdu El Karim Hilali, David Shacham, Reut Frenkel, Olga Abel, Muhammad Abo Abed, Ahmed Abu-Ajaj, Natalia Velikiy, Tamar Freud, Yan Press

**Affiliations:** ^1^Faculty of Health Sciences, Ben-Gurion University of the Negev, Be’er-Sheva, Israel; ^2^Department of Geriatrics, Soroka Medical Center, Be’er-Sheva, Israel; ^3^Siaal Research Center for Family Medicine and Primary Care, Faculty of Health Sciences, Ben-Gurion University of the Negev, Be’er-Sheva, Israel; ^4^Division of Community Health, Faculty of Health Sciences, Ben-Gurion University of the Negev, Be’er-Sheva, Israel; ^5^Unit for Community Geriatrics, Division of Health in the Community, Ben-Gurion University of the Negev, Be’er-Sheva, Israel; ^6^Center for Multidisciplinary Research in Aging, Ben-Gurion University of the Negev, Be’er-Sheva, Israel

**Keywords:** FI-Lab, rehabilitation, mortality, hip fracture, pelvic fracture, predictors, MRFS-R

## Abstract

**Purpose:**

Over the past decade, the Frailty Index based on Laboratory data (FI-Lab index) has been effective in predicting complications during hospitalization, length of hospital stay, changes in functional status, and even mortality. The aims of the present study were to examine the associations between FI-Lab, rehabilitation outcomes, and mortality following hip and pelvic fractures.

**Methods:**

A retrospective study of patients 65 years of age and above who underwent rehabilitation after hip or pelvic fracture in the Geriatric Department, between January 2018 and December 2024. Data included demographic variables, comorbidity, and all-cause mortality. The FI-Lab was calculated based on 26 available blood tests, as well as blood pressure and heart rate measurements. Rehabilitation outcomes were measured using the Montebello Rehabilitation Factor Score-Revised (MRFS-R).

**Results:**

Data were collected for 753 patients. The mean age was 81.9 ± 7.7 years, and 70.3% were women. The mean FI-Lab score was 0.34 ± 0.11. Based on the distribution of FI-Lab scores by quartiles, patients were categorized into four frailty groups: robust (FI-Lab < 0.251), mild (0.252–0.333), moderate (0.334–0.407), and severe (>0.409). No association was found between FI-Lab and MRFS-R as a continuous variable (Spearman *r* = −0.07, *p* = 0.054). A very weak correlation was found between FI-Lab and the length of stay in the Geriatric Department (Spearman *r* = 0.08, *p* = 0.022). After adjusting for age, sex, comorbidity, and complications during hospitalization, patients with higher FI-Lab scores exhibited higher mortality rates. For each 0.01 increase in the FI-Lab score (as a continuous variable), adjusted analyses revealed a 3.6% increase in all-cause mortality within the first year after hospitalization, and a 2.7% increase in all-cause mortality during median follow-up period of 2.2 years.

**Conclusion:**

FI-Lab does not predict rehabilitation success, but does predict overall mortality among patients who underwent rehabilitation after a hip or pelvic fracture.

## Introduction

With the aging of the population, the number of patients suffering from hip and pelvic fractures is increasing (1–3). After these fractures, most patients experience significant functional decline (4–6) and require rehabilitation. In developed countries patients undergo rehabilitation in different settings, including community-based rehabilitation or specialized rehabilitation departments. Identifying factors associated with rehabilitation success is crucial for determining the appropriate rehabilitation setting, planning discharge, and allocating resources (7). Previous studies have identified risk factors associated with rehabilitation outcomes after hip (8, 9) and pelvic fractures (9–11).

Frailty Index based on Laboratory data (FI-Lab) was first proposed by Howlett et al. (12) as an index based solely on routine physical and laboratory tests, serving as a surrogate measure for assessing patient frailty and identifying older adults at increased risk of death. In their study, the authors developed and validated a 23-item FI-LAB index, which included 21 routine blood tests along with measured systolic and diastolic blood pressure. Since then, numerous studies have been conducted using various versions of FI-Lab, comprising between 14 (13) and 77 (14) items. These studies identified FI-Lab as an independent predictor of complications during hospitalization (15, 16), length of hospital stay (17–19), functional decline, higher institutionalization rate after discharge (18, 20), and even increased mortality (13, 14, 17–34). Since FI-Lab can be calculated automatically, based on basic clinical and laboratory data routinely collected in the early stages of hospitalization, theoretically it could be highly useful in decision-making regarding the location and type of rehabilitation following hip and pelvic fractures.

The association between FI-Lab and traditional clinical frailty indices has been examined in studies involving community-dwelling individuals (12, 35–39), patients in long-term care (LTC) settings (40, 41), and hospitalized patients (13, 14, 16, 17, 19, 20, 24, 29, 42), with inconsistent findings. Regarding patients with hip or pelvic fractures specifically, only one study (14) may have included such individuals: among 1,819 patients in a geriatric rehabilitation setting, 48% had musculoskeletal conditions. Unfortunately, the authors did not specify whether this subgroup included patients with hip or pelvic fractures.

Moreover, to the best of our knowledge, no studies have specifically evaluated FI-Lab as a predictor of rehabilitation success following hip or pelvic fractures. Therefore, the aim of this study was to examine the associations between FI-Lab, rehabilitation outcomes, and all-cause mortality in patients aged 65 and older following hip and pelvic fractures.

## Materials and methods

This was a retrospective cross-sectional study based on the electronic medical records (EMR) of patients 65 years and above who underwent rehabilitation after surgical repair for hip fracture or after pelvic fractures in the Geriatric Department of the Soroka University Medical Center (SUMC). All patients in the study had fall-induced fractures, i.e., low-energy trauma.

The department has 25 hospital beds with a multidisciplinary staff including board certified specialists in geriatric medicine, doctors training in geriatric medicine, nurses, physical therapists, occupational therapists, a social worker, and nutritionists. Patients with hip fracture are admitted from the orthopedic department over the first days after the fracture. The process of admission to these departments has been described in the past (43). In short, patients are assessed by geriatricians within the first few days after hip fracture repair. Those who are either unsuitable for or uninterested in community-based rehabilitation, have Health Fund insurance that covers rehabilitation at SUMC, suffer from multiple geriatric syndromes, or prefer rehabilitation in a geriatric department, are transferred there. Patients with pelvic fractures who do not require surgical intervention and meet the admission criteria for the geriatric department (which are the same as those for hip fracture patients) are admitted either directly from the emergency department or from inpatient wards, most commonly orthopedic surgery departments. It is important to emphasize that all hip fracture patients underwent surgical repair, while no pelvic fracture patients did. All patients—both those with femoral neck fractures and those with pelvic fractures—were approved for full weight bearing as tolerated. In the Geriatric Department, both hip fracture patients following surgical repair, and pelvic fracture patients, underwent a comprehensive geriatric assessment followed by the development of an individualized intervention plan.

During their stay, a multidisciplinary team ensured the management of chronic conditions, treatment of complications, nursing care, and rehabilitation. This included physiotherapy five times a week for approximately 45 min per session, occupational therapy several times a week, psychosocial support provided by a social worker, and nutritional intervention by a dietitian. A weekly team meeting was held to review each patient’s progress in the rehabilitation process. The decision to discharge was reached when the patient either achieved their rehabilitation goals (independence in eating, transfers and mobility, and toileting) or reached a functional plateau. Patients discharged to their homes continued to receive multidisciplinary rehabilitation care through dedicated community units followed by transition to rehabilitation clinics. Patients who did not achieve the required level of functional independence were discharged either to a LTC facility or to their homes, based on their own decision or that of their legal guardians.

The present study included male and female patients who underwent rehabilitation in a geriatric department between 1 January 2018, and 31 December 2024, and for whom data were available to assess rehabilitation success on one hand, and sufficient data were available to calculate the FI-Lab index (as described later) on the other.

### Study variables

1. *Sociodemographic variables*: age, sex, level of education, family status, and data on hours of nursing care in the community before the fracture.
2. *Medical variables*: (a) type of fracture, (b) co-morbidity based on the Charlson Comorbidity Index (CCI) (44), and (c) complications during hospitalization in the Geriatric Department such as delirium, pressure sores, venous thromboembolism, and infections. For the analysis, we combined all complications into a single variable, “any complications.” If a patient had multiple complications, such as both delirium and a pressure ulcer, they were counted only once under the “any complications” variable.
3. *Cognitive state variables*: mental state was assessed by the Mini-Mental State Examination (MMSE) (45).
4. *Affective state variable*: patients diagnosed with “depression.”
5. *Functional status variable*: functional status was assessed by the Functional Independence Measure (FIM) (46). This includes anamnestic FIM (anFIM) – FIM of the patient before the current fracture (anamnestic data obtained from the patient and family members), FIM on admission to the Geriatric Department (FIMa), and FIM on discharge from the Geriatric Department (FIMd).
6. *Rehabilitation outcome* was measured with the Montebello Rehabilitation Factor Score-Revised (MRFS-R) (47). The score was calculated according to the following formula:

MRFS-R=((FIMd-FIMa)/FIMd)/((anFIM-FIMa)/anFIM)×100.

The score enabled us to appraise the extent to which patients realized their rehabilitation potential. For example, an MRFS-R score of 56 indicates that the patient achieved 56% of their rehabilitation potential. For the purposes of this study successful rehabilitation was defined as MRFS-R ≥ 50.

In addition, to assess rehabilitation outcomes using a more “classic” approach, we calculated Delta FIM—the difference between the FIM score at discharge and the FIM score at admission (Delta FIM = FIM at discharge − FIM at admission).

1. *FI-LAB construction:* a total of 29 items were used to construct the FI-LAB, including 26 blood tests from venous blood samples and three vital signs recorded during triage in the emergency department: systolic blood pressure, diastolic blood pressure, and heart rate. The laboratory tests used to calculate FI-Lab included hemoglobin, hematocrit, mean corpuscular volume, mean corpuscular hemoglobin, red cell distribution width, white blood cell count, lymphocyte count, and platelet count; creatinine, blood urea, sodium, potassium, chloride, total protein, albumin, total bilirubin, alkaline phosphatase, aspartate aminotransferase (AST), alanine transaminase (ALT), calcium (corrected for albumin level), phosphorus, thyroid-stimulating hormone (TSH), 25-hydroxy vitamin D, parathyroid hormone, vitamin B12, and folic acid levels. Blood tests were performed during evaluations in the emergency department or during the first days of hospitalization. Each item was dichotomized based on the normal reference intervals defined by the SUMC laboratory norms. Values outside the reference range were assigned as a score of 1, while values within the reference range were assigned a score of 0. The FI-Lab score was calculated by summing the number of deficits present and dividing the total by the number of items included. The FI-Lab score ranged from 0 to 1, with higher scores indicating greater frailty. Only patients who had more than 70% of the necessary items (*n* > 21) were included. The FI-LAB scores were categorized by quartiles and as a continuous variable (0.01-point increase).
2. *All-cause mortality* was defined as in-hospital mortality, mortality within 1 year after discharge, and mortality during the entire study period. Data on all-cause mortality was obtained from the SUMC computerized system, which is regularly updated by the Ministry of Interior’s database.

### Statistical analysis

Statistical analyses were conducted using IBM SPSS Statistics, version 29 (SPSS, Inc., Chicago, IL, USA) and R software (Version 4.4.3). A *p*-value < 0.05 was considered statistically significant. Categorical variables are presented as frequencies and percentages. Continuous variables (e.g., age) are described as mean ± SD. A univariate analysis was performed to compare demographic, clinical variables, and rehabilitation outcomes between included vs. not included samples and between hip fracture vs. pelvic fracture samples.

Student’s *t*-test was conducted for continuous variables with a normal distribution. Either the Mann–Whitney *U* test or the Kruskal–Wallis test was used for continuous variables without a normal distribution. The Chi-square test was used for qualitative variables.

For comparisons across FI-Lab groups, the Chi-square test was used for categorical variables, and a *p* for trend was calculated. Spearman’s correlation was used to assess the relationship between FI-Lab scores and other continuous clinical indicators. We applied the Benjamini–Hochberg FDR correction to all exploratory comparisons.

Two Cox regression models were constructed to analyze all-cause mortality, based on frailty status, as assessed by the categorical FI-Lab score: overall mortality at any time and mortality during the first year after admission. These models are presented as survival curves and as adjusted hazard ratios (HRs) for FI-Lab scores, age, gender, CCI scores, and any complications during hospitalization.

Additionally, for the same time frames, two Cox regression models were built to assess all-cause mortality based on frailty status, as measured by the continuous FI-Lab score (0.01-point increase). These models are presented as adjusted HRs for FI-Lab scores, age, gender, CCI scores, and any complications during hospitalization.

The proportional hazards assumption was evaluated for both Cox regression models using Schoenfeld residuals, implemented via the cox.zph() function in R (package survival).

## Results

Over the 6-year follow-up period, 818 patients were admitted to the geriatric department for rehabilitation following a hip or pelvic fracture. Of these, 753 patients had sufficient data to calculate the MRFS-R and were included in the study sample. The average age of 753 patients, included in the study, was 81.9 ± 7.7 years, and 529 (70.3%) were women. Table 1 shows that there were no differences in socio-demographic characteristics, medical status, or in-hospital morbidity between the group of 753 patients included in the sample and the group of 65 patients who were not included. Compared to those not included, a higher proportion of patients in the sample were discharged home at the end of hospitalization (88.4% vs. 70.8%), and more patients in this group were alive at the end of the follow-up period (64.0% vs. 40.0%). Of the 753 patients included in the sample, 568 underwent rehabilitation after hip fracture repair, and 185 after pelvic fracture. Supplementary Table 1 presents the data for all 753 patients, along with their distribution by group. The only notable differences between the groups was a higher rate of any complications in the hip fracture group (58.6%) compared to the pelvic fracture group (41.6%), *p* = 0.003. Other socio-demographic and clinical characteristics did not differ significantly between the groups.

**TABLE 1:** Socio-demographic and clinical characteristics: comparison between patients who were included in the study and those who were not.

Variables	All sample patients (*N* = 753)	Not included in the sample (*N* = 65)	*P*
**Socio-demographic**			
Age (years) (mean ± SD)	81.9 ± 7.7	84.1 ± 8.3	0.271
Gender (female) [*n* (%)]	529 (70.3)	46 (70.8)	0.999
Family status (married) [*n* (%)]	317 (42.1)	19 (29.2)	0.220
Nursing caregiver (yes) [*n* (%)] (miss = 43)	438 (61.7)	36 (64.3)	0.925
Education > 12 years [*n* (%)] (miss = 15)	229 (31.0)	17 (28.8)	0.863
**Medical status**			
CCI (mean ± SD)	4.1 ± 2.9	4.3 ± 3.1	0.829
MMSE (mean ± SD) (miss = 101)	23.2 ± 5.3	21.5 ± 4.9	0.179
Depression [*n* (%)]	71 (9.4)	11 (16.9)	0.267
BMI (mean ± SD) (miss = 12)	25.2 ± 4.5	24.8 ± 4.8	0.711
**Morbidity during rehab process [*n* (%)]**			
Delirium	123 (16.3)	10 (15.4)	0.999
Any infection	194 (25.8)	20 (30.8)	0.725
Cardiovascular (IHD, HF, stroke)	66 (8.8)	7 (10.8)	0.866
Thromboembolism	18 (2.4)	0 (0.0)	0.905
Pressure ulcers	20 (2.7)	3 (4.6)	0.769
Any complication	410 (54.4)	36 (55.4)	0.999
Hospitalization duration (days) (mean ± SD)	19.9 (8.9)	19.9 ± 8.9	0.660
**Functional status**			
anFIM (mean ± SD)	109.2 ± 15.5	NA	
FIMa (mean ± SD)	62.0 ± 17.8	NA	
FIMd (mean ± SD)	85.5 ± 18.9	NA	
MRFS-R (mean ± SD) (median)	53.5 ± 79.7 (65.7)	NA	
Patients with MRFS-R ≥ 50 [*n* (%)]	523 (69.5)	NA	
Delta FIM (mean ± SD)	23.5 ± 19.5	NA	
**FI-Lab**			
Robust < 0.251	181 (24.0)	17 (26.2)	0.716
Mildly frail (0.252–0.333) (*N* = 188)	188 (25.0)	19 (29.2)	
Moderately frail (0.334–0.407) (*N* = 185)	185 (24.6)	10 (15.4)	
Severely frail > 0.407 (*N* = 199)	199 (26.4)	19 (29.2)	
FI-Lab (mean ± SD)	0.34 ± 0.11	0.35 ± 0.13	0.828
**Rehabilitation outcome [*n* (%)]**			
Discharged to home	663 (88.4)	46 (70.8)	0.0018
Transferred to other hospital departments	53 (7.1)	7 (10.8)	
Discharged to LTC	34 (4.5)	3 (4.6)	
In-hospital mortality	3 (0.4)	9 (13.9)	
**All-cause mortality [*n* (%)]**			
Alive	482 (64.0)	26 (40.0)	0.0018
Died over the first 30 days after admission (including in-hospital)	10 (1.3)	11 (16.9)	
Died between 30 days and 1 year	82 (10.9)	6 (9.2)	
Died after a year or more	179 (23.8)	22 (33.9)	

CCI, the Charlson Comorbidity Index; MMSE, the Mini-Mental State Examination; BMI, body mass index; IHD, ischemic heart disease; HF, heart failure; FIM, the Functional Independence Measure; anFIM, anamnestic FIM; FIMa, FIM on admission; FIMd, FIM on discharge; MRFS-R, the Montebello Rehabilitation Factor Score-Revised; LTC, long-term care; NA, not applicable.

### Frailty index based on laboratory data (FI-Lab)

Data on the variables included in FI-Lab, are presented in Supplementary Table 2. As can be seen, there were no differences in any variable that includes FI-LAB between the groups that were included and not included in the sample, nor between the hip fracture and pelvic fracture groups.

All patients in the sample (and 65 patients who were not included in the sample) had at least 21 available components for calculating FI-Lab. The average FI-Lab score for the 753 patients was 0.34 ± 0.11. Patients were divided into four quartiles based on the FI-Lab distribution (Table 1):

• 181 patients (24.0%) – FI-Lab score below 0.251 (“Robust” group)
• 188 patients (25.0%) – FI-Lab score between 0.252 and 0.333 (“Mildly Frail” group)
• 185 patients (24.6%) – FI-Lab score between 0.334 and 0.407 (“Moderately Frail” group)
• 199 patients (26.4%) – FI-Lab score above 0.407 (“Severely Frail” group)

Frailty Index based on Laboratory data was not normally distributed, and several patients had identical FI-LAB scores. For this reason, the number of patients in each quartile was not equal.

Table 1 shows that there was no difference in the average FI-Lab score between the groups that were included and not included in the sample. Additionally, within the 753 patients included in the study, no significant difference was found in the average FI-Lab score between the 568 patients with hip fractures and the 185 patients with pelvic fractures (Supplementary Table 1).

Furthermore, no differences were found between the groups that were included and not included in the sample (Table 1), as well as between the hip fracture and pelvic fracture groups (Supplementary Table 1), in terms of the proportion of patients classified as robust, mildly frail, moderately frail, or severely frail.

### Association between FI-Lab, socio-demographic and clinical variables, and successful rehabilitation

Table 2 presents the correlations between FI-Lab and the continuous socio-demographic and clinical characteristics of the 753 patients included in the study. The results show a weak positive correlation between FI-Lab and age (*r* = 0.09, *p* = 0.003), CCI (*r* = 0.273, *p* = 0.0007), and length of stay in the Geriatric Department (*r* = 0.083, *p* = 0.038). A weak negative correlation was found between FI-Lab and MMSE score (*r* = −0.105, *p* = 0.024). No correlation was found between MRFS-R and FI-Lab (*r* = −0.07, *p* = 0.075). No association was found between FI-Lab and the absolute improvement in FIM during rehabilitation in the geriatric ward (Delta FIM) (*r* = −0.059, *p* = 0.122).

**TABLE 2:** Correlation between FI-Lab and the patients’ socio-demographic and clinical characteristics.

Variable	Total sample patients (*N* = 753)		
	*N*	Spearman *r*	*P*
Age, years	753	0.09	0.003
Body mass index	741	−0.017	0.645
MMSE	652	−0.105	0.024
CCI	753	0.273	0.0007
LoS (days)	753	0.083	0.038
MRFS-R	753	−0.07	0.075
Delta FIM	753	−0.059	0.122

CCI, the Charlson Comorbidity Index; MMSE, the Mini-Mental State Examination; LoS, length of stay in the geriatric department; MRFS-R, the Montebello Rehabilitation Factor Score-Revised; FIM, the Functional Independence Measure.

Supplementary Table 3 shows that no correlation was found between FI-LAB and socio-demographic or clinical variables among patients with hip fractures. Among patients with pelvic fractures, only the CCI score was correlated with FI-LAB as a continuous variable (*r* = 0.335, *p* = 0.007). In both groups (hip fractures and pelvic fractures), no correlation was found between FI-LAB as a continuous variable and rehabilitation outcomes (MRFS-R or Delta FIM).

The association between dichotomous variables and FI-Lab groups in the cohort of 753 patients included in the sample is presented in Table 3.

**TABLE 3:** The association between dichotomous variables and FI-Lab groups (*N* = 753).

Variables	Frailty status group by FI-LAB score								*P* (for trend)
	Robust (*N* = 181)		Mildly frail (*N* = 188)		Moderately frail (*N* = 185)		Severely frail (*N* = 199)		
	*N*	%	*N*	%	*N*	%	*N*	%	
**Socio-demographic**									
Gender (female) [*n* (%)]	147	81.2	136	72.3	126	68.1	120	60.3	0.004
Family status (married) [*n* (%)]	75	41.4	85	45.2	70	37.8	87	43.7	0.389
Nursing caregiver (yes) [*n* (%)] (mis = 43)	99	57.6	104	59.1	106	60.6	129	69.0	0.069
**Medical status**									
Depression [*n* (%)]	19	10.5	14	7.4	22	11.9	16	8.0	0.747
Delirium [*n* (%)]	25	13.8	29	15.4	30	16.2	39	19.6	0.204
Any complication [*n* (%)]	76	42	103	54.8	107	57.8	120	60.3	0.004
**Functional status**									
Patients with MRFS-R ≥ 50 [*n* (%)]	133	73.5	136	72.3	124	67	69	65.3	0.094
Discharged to LCT [*n* (%)]	10	5.7	13	7.2	4	2.4	7	4.1	0.231

MRFS-R, the Montebello Rehabilitation Factor Score-Revised; LTC, long-term care; mis, missing.

The proportion of women decreased with increasing frailty levels (*p* for trend 0.004). As frailty severity increased, more patients experienced a higher rate of complications (*p* for trend 0.004) during hospitalization in the Geriatric Department.

Additionally, the proportion of patients with MRFS-R, which was defined in this study as “successful rehabilitation,” decreased as frailty severity increased—from 73.5% in the robust group to 65.3% in the frail group—but this difference did not reach statistical significance (*p* for trend = 0.094). No association was found between frailty severity according to FI-Lab and the proportion of patients transferred to LTC at the end of rehabilitation in the Geriatric Department.

As shown in Supplementary Table 4, among patients with hip fractures, the proportion of women decreased with increasing frailty severity (*p* for trend < 0.001). Among patients with pelvic fractures, a higher level of frailty was associated with a greater likelihood of having been under the care of a nursing caregiver prior to the fracture (*p* for trend = 0.009). In addition, increased frailty severity in the pelvic fracture group was associated with a higher likelihood of developing delirium (*p* for trend = 0.016) and experiencing any complication during hospitalization (*p* for trend = 0.005). In both subgroups—hip fracture and pelvic fracture—no association was found between the level of frailty and rehabilitation outcomes or the likelihood of being discharged to LTC.

### Association between FI-Lab and all-cause mortality

The follow-up period for patients included in the sample ranged from 3 to 2,459 days, with an average of 848.0 ± 660.7 days and a median of 808.5 days. During this period, 271 patients (36.0%) passed away and 482 (64.0%) remained alive.

The patients who died were older, included a higher proportion of men, and had a higher incidence of any complications during their hospitalization in the Geriatric Department. Additionally, they had a higher CCI score and a higher FI-Lab score (see Table 4). Also, within the patient subgroups (hip fracture and pelvic fracture—Supplementary Table 5), those who did not survive until the end of the study had higher FI-LAB scores.

**TABLE 4:** All-cause mortality during the follow-up period.

Variables	Alive (*N* = 482)	Dead (*N* = 271)	P
Age (years) (mean ± SD)	80.8 ± 7.5	83.8 ± 7.6	<0.0001
Gender (female) [*n* (%)]	363 (75.3)	166 (61.3)	<0.0001
Depression (yes) [*n* (%)]	50 (10.4)	21 (7.7)	0.298
BMI (mean ± SD) (miss = 12)	25.2 ± 4.5	25.2 ± 4.5	0.956
MMSE (mean ± SD)	23.3 ± 5.3	22.8 ± 5.4	0.247
Any complication (yes) [*n* (%)]	247 (51.2)	163 (60.2)	0.022
CCI (mean ± SD)	3.7 ± 2.7	4.7 ± 3.1	<0.0001
MRFS-R (mean ± SD)	57.4 ± 84.0	46.6 ± 71.1	0.073
Delta FIM	24.6 ± 18.7	21.5 ± 20.7	0.032
FI-Lab (mean ± SD)	0.32 ± 0.11	0.37 ± 0.11	<0.0001

CCI, the Charlson Comorbidity Index; MMSE, the Mini-Mental State Examination; BMI, body mass index; MRFS-R, the Montebello Rehabilitation Factor Score-Revised.

Variables that were found to be significant in the univariate analysis were included in the Cox models (see Table 5).

**TABLE 5:** Cox proportional hazard models for all-cause mortality.

Model	Variables	All-cause mortality during the first year				All-cause mortality during entire follow-up period			
		Hazard ratio	95.0% CI		*P*	Hazard ratio	95.0% CI		*P*
			Lower	Upper			Lower	Upper	
1	FI-LAB (groups)								
	Robust (reference)	1.000				1.000			
	Mildly frail	1.666	0.710	3.910	0.241	1.144	0.766	1.710	0.511
	Moderately frail	2.38	1.062	5.333	0.035	1.499	1.024	2.195	0.037
	Severely frail	3.802	1.744	8.289	<0.001	2.176	1.508	3.139	<0.001
	Gender (female)	0.644	0.422	0.982	0.041	0.732	0.568	0.943	0.016
	Age (years)	1.028	1.000	1.057	0.047	1.042	1.025	1.059	<0.001
	Any complication	2.083	1.300	3.336	0.002	1.555	1.213	1.993	<0.001
	CCI	1.037	0.967	1.111	0.310	1.097	1.052	1.143	<0.001
2	FI-LAB (continuous)	1.036	1.017	1.056	<0.001	1.027	1.016	1.038	<0.001
	Gender (male)	0.632	0.414	0.966	0.034	0.731	0.567	0.941	0.015
	Age (years)	1.030	1.002	1.058	0.036	1.042	1.026	1.059	<0.001
	Any complication	2.081	1.297	3.339	0.002	1.551	1.209	1.989	<0.001
	CCI	1.033	0.963	1.108	0.369	1.094	1.049	1.141	<0.001

CCI, the Charlson Comorbidity Index.

The proportional hazards assumption was assessed for both Cox regression models using Schoenfeld residuals (cox.zph() function in R). For the model with FI-LAB as a categorical variable, no significant violations were observed for most covariates (all *p* > 0.05), and the global test was non-significant (*p* = 0.066), indicating that the overall model satisfied the proportional hazards assumption. One covariate, any complication, had a borderline result (*p* = 0.046), suggesting a possible deviation; however, given the non-significant global test and consistent findings across models, we retained this variable in the model without modification. For the second model, which included FI-LAB as a continuous variable, the proportional hazards assumption was fully met for all covariates (all *p* > 0.05), including FI-LAB (*p* = 0.723), and the global test was clearly non-significant (*p* = 0.572). These findings support the validity of both models under the proportional hazards assumption.

#### Model 1: Cox proportional hazard models for all-cause mortality (FI-Lab categorized by groups)

In Model 1, where FI-Lab was analyzed as a categorical variable, we found that, compared to robust patients, the risk of all-cause mortality within the *first year* after admission to the Geriatric Department was higher in patients with moderate (HR = 2.38; 95% CI: 1.062–5.333, *p* = 0.035) and severe (HR = 3.802; 95% CI: 1.744–8.289, *p* < 0.001) frailty. Other independent predictors of 1-year mortality included age (HR = 1.028; 95% CI: 1.000–1.057, *p* = 0.047), female gender (HR = 0.644; 95% CI: 0.422–0.982, *p* = 0.041) and the presence of any complications during hospitalization in the geriatric department (HR = 2.083; 95% CI: 1.300–3.336, *p* = 0.002).

Compared to robust patients, the risk for mortality over the *entire follow-up period* was higher in patients with moderate (HR = 1.499; 95% CI: 1.024–2.195, *p* = 0.037) and severe (HR = 2.176; 95% CI: 1.508–3.139, *p* < 0.001) frailty. Additionally, age (HR = 1.042; 95% CI: 1.025–1.059, *p* < 0.001), female gender (HR = 0.732; 95% CI: 0.568–0.943, *p* = 0.016), CCI score (HR = 1.097; 95% CI: 1.052–1.143, *p* < 0.001), and any complications during hospitalization in the Geriatric Department (HR = 1.155; 95% CI: 1.213–1.993, *p* < 0.001) were also identified as independent predictors of all-cause mortality throughout *the hole follow-up period*.

#### Model 2: Cox proportional hazard models for all-cause mortality (FI-Lab as a continues variable)

For every 0.01-point increase in FI-Lab, all-cause mortality increased by 3.6% during *the first year* of follow-up. In this model, an independent inverse association was found between female gender and 1-year mortality, while an independent direct association was observed between mortality, advanced age, and any complications during hospitalization in the geriatric department.

Independent predictors of increased all-cause mortality over *the entire follow-up period* included FI-Lab (every 0.01-point increase was associated with a 2.7% increase in mortality), advanced age, male gender, CCI score, and any complications during hospitalization in the geriatric department.

**FIGURE 1:**
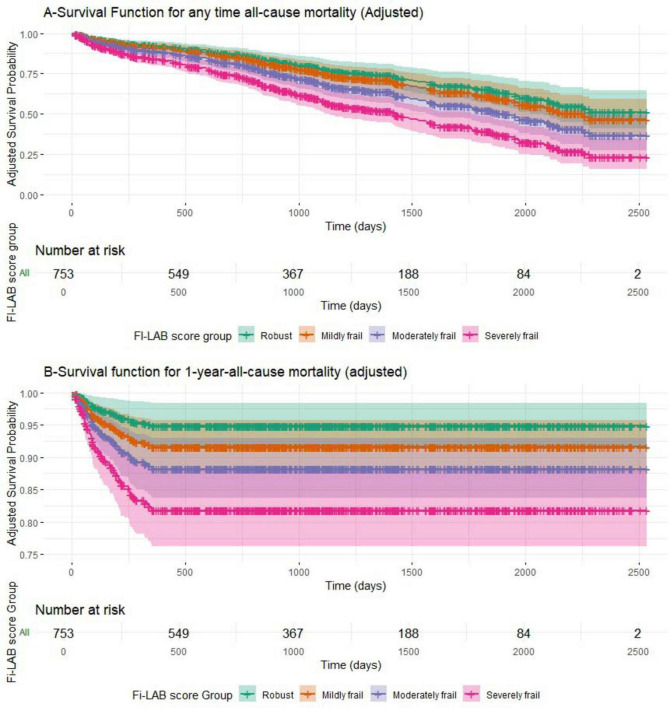
**(A,B)** Survival function for all-cause mortality.

Figure 1 presents the adjusted survival curves stratified by FI-Lab-based frailty status for the entire follow-up period [A] and for 1-year follow-up [B], based on the multivariable Cox models (see Table 5). A consistent gradient was observed, with progressively lower survival probabilities in higher frailty groups (*p* < 0.001 for all comparisons). Confidence intervals are displayed around the survival estimates, and the number of patients at risk is shown at predefined time points to enhance interpretability.

#### Cox proportional hazards models for all-cause mortality in subgroups

Since only 19 out of 185 patients in the pelvic fracture group died during the first year, dividing them into four groups was not feasible, and therefore model 1 could not be constructed for this group. Among patients with hip fractures, those with severe frailty had higher risk of death during the *first year* compared to robust patients (HR = 2.748; 95% CI: 1.235–6.115, *p* = 0.013). According to model 2, the risk of death during the *first year* increased by 3.1% for every 0.01-point increase in FI-LAB among patients with hip fractures. In the group of patients with pelvic fractures, each 0.01-point increase in the FI-LAB score was associated with a 6.1% increase in *1-year mortality* following hospitalization (see Supplementary Table 6).

According to model 1, mortality over the *entire follow-up period* (Supplementary Table 7) was higher among patients with severe frailty compared to robust patients—both in the hip fracture group (HR = 1.933; 95% CI: 1.283–2.913, *p* = 0.002) and in the pelvic fracture group (HR = 4.14; 95% CI: 1.779–9.632, *p* < 0.001). According to model 2, when FI-LAB was treated as a continuous variable, each 0.01-point increase in the FI-LAB score was associated with a 2.3% increase in mortality over the *entire follow-up period* in the hip fracture group, and a 4.7% increase in mortality in the pelvic fracture group.

## Discussion

The current study had two objectives: to examine the association between FI-Lab and rehabilitation outcomes and between FI-Lab and mortality.

### FI-Lab and rehabilitation outcomes

Whether rehabilitation success was measured as a continuous MRFS-R score (Table 2) or as a dichotomous variable (MRFS-R score ≥ 50%; Table 3), no association was found between FI-Lab and rehabilitation success.

Since there is a possibility that MRFS-R, despite being examined in several of our previous studies (11, 43, 48–50), may not be the most optimal measure of rehabilitation success, we decided to evaluate an alternative rehabilitation measure: Delta FIM (FIM at discharge – FIM at admission). Here too, no association was found between FI-Lab and Delta FIM (Spearman *r* = −0.059, *p* = 0.122). It is important to note that even in the subgroup analysis of patients with hip fractures and pelvic fractures, no association was found between FI-LAB and rehabilitation success.

To our knowledge, this is the first study to examine the association between FI-Lab and rehabilitation success. In our literature review, we found only one study conducted among patients hospitalized in a geriatric rehabilitation setting, with two published articles based on its findings (14, 51). Only one of these articles (14) examined rehabilitation outcomes, and no association was found between FI-Lab and functional decline or institutionalization. Unfortunately, in this study, the researchers did not use the FIM measure, making it impossible to directly compare their results with ours.

The association between FI-Lab and institutionalization, functional status, and healthcare resource utilization has been examined in a limited number of studies. Our literature review identified only four studies that addressed the relationship between FI-Lab and institutionalization, two conducted in community settings (12, 37), one focusing on veterans in the United States (20), and another on patients visiting the emergency department (18). All these studies found a higher rate of institutionalization among patients with higher FI-Lab scores.

In the study by Blodgett et al. (52), conducted in a community setting, a higher FI-Lab score was associated with a higher rate of functional decline and greater use of healthcare resources. In a study of acutely hospitalized older patients, Nakashima et al. (19) found an association between FI-Lab and basic and instrumental functioning, approximately 2 weeks before hospital admission. In another study by the same authors (39), among patients starting home-based medical care, an association was found between basic functioning and FI-Lab. However, since these studies were not conducted among rehabilitation patients, any comparison of their findings to those of the current study is of limited value.

In our opinion, there are several reasons why no association was found between FI-LAB and successful rehabilitation. Rehabilitation success depends on multiple factors, including cognitive and affective status, comorbidity, level of family support, and motivation. These variables are likely not directly reflected in laboratory test results or in blood pressure and pulse measurements—the data on which the FI-Lab index is based.

Another possible explanation for our findings is that FI-Lab may only capture certain aspects of frailty. It is important to note that previous studies examining the relationship between FI-Lab and other frailty indices have yielded conflicting results (12–14, 17, 19, 20, 24, 29, 35–42, 53).

Of course, it is also possible that the rehabilitation period (in our case, approximately 3 weeks) is too long for a continuous association between FI-Lab, measured at the beginning of hospitalization, and rehabilitation success, especially considering that the MRFS-R formula includes FIM at discharge. Jäger et al. (25) found that the FI-Lab score fluctuates during hospitalization and that FI-Lab at admission was a weaker predictor of mortality at 6 months and 1 year post-discharge compared to FI-Lab at discharge. It is very possible that to observe a meaningful association between FI-Lab and rehabilitation outcomes, FI-Lab should be measured not only at the beginning of rehabilitation but also at later stages.

Ultimately, a large, multicenter, prospective study with repeated FI-LAB assessments and simultaneous evaluation of other frailty models, conducted specifically among a homogeneous rehabilitation population, would likely be necessary to determine whether a pre-rehabilitation FI-LAB score is relevant for predicting rehabilitation outcomes.

### FI-Lab and all-cause mortality

Frailty Index based on Laboratory data was associated with increased mortality, as a continuous variable and as a categorical variable. These findings are consistent with numerous other studies (13, 14, 17, 19–21, 24, 25, 30, 34, 51, 54, 55) that examined the association between FI-Lab in older hospitalized patients and mortality. Clinical deficits represent the manifestation of unrepaired and/or unresolved damage at the subcellular, tissue, and organ levels (12, 37). The more pronounced these impairments are, the less favorable the prognosis. The aggregation of subclinical deficits, reflected in laboratory abnormalities, into a frailty index—even when individual deficits are not directly associated with mortality risk—has nonetheless been shown to be significantly associated with mortality, independently of a clinical frailty index (12, 38, 40). FI-LAB, which is based on laboratory tests, may reflect impaired repair processes that play a significant role in a wide range of age-related diseases (40).

It should also be noted that among patients with pelvic fractures, each 0.01-point increase in FI-LAB was associated with a higher HR for mortality—both during the first year after hospitalization and over the entire follow-up period—compared to the HR observed among patients with hip fractures (Supplementary Tables 6, 7). Considering that the two groups did not differ in their socio-demographic or clinical characteristics (aside from a higher rate of complications among patients with hip fractures), and that their FI-LAB scores were also similar, the question arises as to why the association between FI-LAB and mortality differs between the two populations. Could it be that other variables—such as comorbidities not captured by the CCI—were not accounted for and may influence both FI-LAB and mortality? Or is it possible that in the pelvic fracture group, FI-LAB is a more predictive marker of mortality for other reasons?

Based on accumulated data, including the current study, it can be concluded that FI-Lab at the beginning of hospitalization can serve as a fairly reliable predictor of future mortality. However, despite the availability and automatic calculability of this index, we support the recommendation of Nakashima et al. (39) not to rely solely on FI-Lab calculations but to proceed with a comprehensive geriatric assessment. Relying exclusively on FI-Lab may lead physicians to incorrect conclusions regarding treatment planning and hospital discharge decisions.

Our study has several strengths. To our knowledge, this is the first study to examine the association between FI-Lab and rehabilitation outcomes in older adults undergoing rehabilitation after a hip or pelvic fracture. The study includes a relatively large sample of over 750 patients, with a median follow-up time of 6 years. Additionally, the study was conducted in a Geriatric Department that has maintained consistent standards and routine procedures, including cognitive and functional assessments, throughout the years. Furthermore, mortality data in our study are based on reliable and up-to-date records.

However, the study also has several notable limitations. The study population was heterogeneous, including both hip and pelvic fracture patients. In the subgroup analysis of pelvic and hip fractures, these groups appear to behave similarly in terms of FI-LAB and its association with rehabilitation outcomes and mortality. However, it should be noted that these are not homogeneous groups (for example, patients with hip fractures underwent surgical repair, while those with pelvic fractures did not), and therefore, the study findings should be interpreted with caution.

A key limitation of this study is its retrospective design, which restricted our ability to control for unmeasured confounders such as variability in rehabilitation interventions, family support, and socioeconomic status. Additionally, 65 patients were excluded due to missing FIM data. Although these patients represented a small proportion of the total cohort, they had significantly higher early mortality and were less likely to be discharged home, suggesting greater clinical vulnerability and raising the possibility of survival bias. Baseline socio-demographic and medical characteristics, including age, comorbidity burden, cognitive function, and FI-Lab scores, were largely comparable between included and excluded patients. However, the absence of detailed rehabilitation outcome data for the excluded group precluded a formal sensitivity analysis. This limitation has been acknowledged and its potential impact on the results should be considered when interpreting the findings. Finally, as the study was conducted in a single medical center in one country, its findings cannot be generalized to the entire population of rehabilitation patients.

## Conclusion

Frailty Index based on Laboratory data did not predict rehabilitation outcomes following a hip or pelvic fracture in an older population. As shown in previous studies, FI-Lab was associated with mortality. A large-scale prospective study is needed to determine whether FI-Lab at admission to the rehabilitation department can be reliably used as a predictor of successful rehabilitation.

## Data Availability

The datasets generated and analyzed during the current study are not publicly available due to legal and data security reasons, but are available from the corresponding author on reasonable request. Requests to access these datasets should be directed to Yan Press, yanp@bgu.ac.il.
