# Supplementary material for: FI-Lab predicts all-cause mortality, but not successful rehabilitation, among patients aged 65 and older after hip or pelvic fracture
Source: Front Med (Lausanne). 2025 Jul 16;12:1627026. doi: 10.3389/fmed.2025.1627026 (PMC12307480; doi:10.3389/fmed.2025.1627026)
Supplement: Supplementary file 1 [file Table_1.docx]

**Supplement Table 1.** **Socio-demographic and clinical characteristics: Comparison between patients with hip and pelvic fractures.**

|  | **Hip fracture (N=568)** | **Pelvic fracture (N=185)** | **P** |
| --- | --- | --- | --- |
| **Socio-demographic** |  |  |  |
| Age (years) (Mean±SD) | 82.0±7.8 | 81.5±7.4 | 0.527 |
| Gender (female) [n (%)] | 387 (68.1) | 142 (76.8) | 0.203 |
| Family status (married) [n (%)] | 249 (43.8) | 68 (36.8) | 0.311 |
| Nursing caregiver (yes) [n (%)] (miss=43) | 328 (61.7) | 110 (61.8) | 0.999 |
| Education >12 years [n (%)] (miss=15) | 167 (29.9) | 62 (34.4) | 0.494 |
| **Medical status** |  |  |  |
| CCI (Mean±SD) | 4.1±2.8 | 4.2±3.2 | 0.746 |
| MMSE (Mean±SD) (miss=101) | 23.0±5.4 | 23.8±4.9 | 0.316 |
| Depression [n (%)] | 47 (8.3) | 24 (13.0) | 0.273 |
| BMI, (Mean±SD) (miss=12) | 25.1±4.5 | 25.4±4.6 | 0.701 |
| **Morbidity during rehab process [n (%)]** |  |  |  |
| Delirium | 103 (18.3) | 20 (10.8) | 0.203 |
| Any infection | 159 (28.0) | 35 (18.9) | 0.203 |
| Cardiovascular (IHD, HF, stroke) | 51 (9.0) | 15 (8.1) | 0.877 |
| Thromboembolism | 16 (2.8) | 2 (1.1) | 0.511 |
| Pressure Ulcers | 18 (3.2) | 2 (1.1) | 0.395 |
| Any complication | 333 (58.6) | 77 (41.6) | 0.003 |
| Hospitalization duration (days)(Mean±SD) | 20.1 (9.2) | 19.2 (8.1) | 0.542 |
| **Functional status** |  |  |  |
| anFIM (Mean±SD) | 108.7±16.1 | 110.9±13.4 | 0.513 |
| FIMa (Mean±SD) | 61.7±17.8 | 62.7±17.9 | 0.524 |
| FIMd (Mean±SD) | 84.8±19.0 | 87.5±18.5 | 0.203 |
| MRFS-R (Mean±SD) (Median) | 53.3±66.8 (65.3) | 54.2±110.5 (67.9) | 0.395 |
| Patients with MRFS-R ≥50 [n (%)]  Delta FIM (Mean±SD) | 392 (69.0)  23.1±19.1 | 131 (70.8)  24.8±20.7 | 0.769  0.662 |
| **FI-Lab** |  |  |  |
| Robust < 0.251 | 127 (22.4) | 54 (29.2) | 0.316 |
| Mildly frail (0.252-0.333) (N=188) | 138 (24.3) | 50 (27.0) |  |
| Moderately frail (0.334 -0.407) (N=185) | 146 (25.7) | 39 (21.1) |  |
| Severely Frail >0.407 (N=199) | 157 (27.6) | 42 (22.7) |  |
| FI-Lab (Mean±SD) | 0.34±0.11 | 0.32±0.12 | 0.203 |
| **Rehabilitation outcome [n (%)]** |  |  |  |
| Discharged to home | 495 (87.6) | 168 (90.8) | 0.524 |
| Transferred to other hospital departments | 44 (7.8) | 9 (4.9) |  |
| Discharged to LTC | 26 (4.6) | 8 (4.3) |  |
| In-hospital mortality | 3 (0.4) | 0 (0.0) |  |
| **All-cause mortality [n (%)]** |  |  |  |
| **Alive** | 355 (62.5) | 127 (68.7) | 0.524 |
| **Died over the first 30 days after**  **admission (including in-hospital)** | 9 (1.6) | 1 (0.6) |  |
| **Died between 30 days and one year** | 64 (11.3) | 18 (9.7) |  |
| **Died after a year or more** | 140 (24.7) | 39 (21.1) |  |

CCI- the Charlson Comorbidity Index; MMSE-the Mini-Mental State Examination; BMI - body mass index; IHD - ischemic heart disease; HF - heart failure; FIM- the Functional Independence Measure; anFIM- anamnestic FIM; FIMa-FIM on admission; FIMd- FIM on discharge; MRFS-R – the Montebello Rehabilitation Factor Score- Revised ; LTC- long-term care

**Supplement Table 2: Components of the FI-Lab Index: Means and Proportion of Abnormal Test Results**

|  | **Patients, included in the sample (N=753)** | **Patients, not included in the sample (N=65)** | **P*** | **Patients, included in the sample with hip fracture (N=568)** | **Patients, included in the sample with pelvic fracture (N=185)** | **P**** |
| --- | --- | --- | --- | --- | --- | --- |
| **FI-Lab (Mean±SD)** | | | | | | |
| Systolic Blood Pressure (mmHg) | 151.4±25.7 | 146.1±28.4 | 0.702 | 151.7±25.4 | 150.6±26.5 | 0.918 |
| Diastolic Blood Pressure (mmHg) | 75.2±16.3 | 72.7±17.7 | 0.785 | 74.1±15.9 | 75.5±1.3 | 0.999 |
| Heart rate (beats/min) | 81.2±15.4 | 81.7±14.9 | 0.995 | 81.3±15.5 | 81.0±15.3 | 0.918 |
| Hemoglobin (g/dl) | 12.1±1.8 | 12.2±2.1 | 0.995 | 12.0±1.8 | 12.3±1.7 | 0.356 |
| Hematocrit (%) (miss=26) | 37.1±5.3 | 37.4±5.9 | 0.995 | 36.9±5.3 | 37.6±5.1 | 0.359 |
| Mean corpuscular volume (FL) | 89.2±7.2 | 89.6±5.2 | 0.995 | 89.2±7.1 | 89.1±7.3 | 0.999 |
| Mean corpuscular Hemoglobin (pg) | 29.1±2.7 | 29.2±2.0 | 0.995 | 29.1±2.6 | 29.1±2.9 | 0.999 |
| Red Cell Distribution Width (%) (miss=36) | 15.3±1.9 | 15.3±1.6 | 0.785 | 15.4±1.9 | 15.1±1.8 | 0.356 |
| White blood cells (*10^3^ /ul) | 11.1±4.0 | 10.6±4.6 | 0.788 | 11.3±4.0 | 10.6±3.6 | 0.261 |
| Lymphocytes (*10^3^ /ul) (miss=1) | 1.3±1.2 | 1.2±0.6 | 0.995 | 1.3±0.9 | 1.4±1.7 | 0.918 |
| Platelets *10^3^ /ul | 223.9±79.8 | 217.6±120.4 | 0.702 | 227.7±81.4 | 212.4±73.5 | 0.356 |
| Creatinine (mg/dL) | 1.2±1.2 | 1.2±0.9 | 0.785 | 1.2±1.2 | 1.2±1.2 | 0.382 |
| Blood urea (mg/dL) | 54.3±28.4 | 61.6±39.1 | 0.702 | 55.0±28.9 | 52.3±26.9 | 0.457 |
| Sodium (mEq/L) | 138.0±3.9 | 138.4±3.6 | 0.988 | 137.9±3.8 | 138.1±4.2 | 0.371 |
| Potassium (mEq/l) | 4.3±0.6 | 4.3±0.6 | 0.995 | 4.3±0.6 | 4.3±0.5 | 0.709 |
| Chloride (mEq/L) (miss=26) | 102.6±4.7 | 102.5±3.9 | 0.995 | 102.6±4.7 | 102.7±4.7 | 0.918 |
| Protein, total (g/L) (miss=6) | 6.0±0.7 | 6.0±0.7 | 0.995 | 6.0±0.7 | 6.1±0.7 | 0.356 |
| Albumin (g/L) (miss=5) | 3.4±0.4 | 3.3±0.4 | 0.702 | 3.3±0.4 | 3.4±0.4 | 0.232 |
| Bilirubin Total (mg/dL) (miss=27) | 0.7±0.4 | 0.8±0.4 | 0.702 | 0.7±0.4 | 0.7±0.4 | 0.371 |
| Phosphatase, alkaline (U/L) | 95.9±63.8 | 91.7±38.9 | 0.995 | 95.5±67.0 | 97.2±54.3 | 0.999 |
| AST (U/L) (miss=1) | 28.9±27.3 | 28.1±14.1 | 0.882 | 28.7±28.5 | 29.6±23.3 | 0.371 |
| ALT (U/L) (miss=1) | 19.8±24.2 | 17.3±6.7 | 0.995 | 19.4±26.0 | 21.0±17.5 | 0.232 |
| Calcium (corrected) (mg/dL) (miss=1) | 9.7±0.6 | 9.6±0.6 | 0.702 | 9.7±0.6 | 9.7±0.6 | 0.918 |
| Phosphorus, inorganic (mg/dL) (miss=2) | 3.3±0.8 | 3.5±0.9 | 0.586 | 3.3±0.8 | 3.4±0.8 | 0.356 |
| TSH (μIU/L) (miss=21) | 2.9±3.6 | 3.3±4.4 | 0.995 | 2.8±3.7 | 3.0±2.9 | 0.261 |
| Vitamin D (miss=97) | 53.2±24.7 | 47.0±21.2 | 0.702 | 53.0±24.1 | 54.7±26.7 | 0.890 |
| PTH (pg/mL) (miss=116) | 105.0±129.9 | 116.8±93.9 | 0.702 | 105.5±116.4 | 103.3±166.2 | 0.356 |
| Vitamin B12 (pg/L) (miss=42) | 526.2±343.4 | 591.2±452.0 | 0.995 | 528.8±346.3 | 517.9±334.7 | 0.918 |
| Folic acid (ng/mL) (miss=84) | 7.1±4.6 | 6.6±4.6 | 0.881 | 7.1±4.5 | 7.4±4.6 | 0.747 |
| **FI-LAB positive (n (%))** | | | | | | |
| Systolic Blood Pressure | 510 (67.7) | 39 (60.0) | 0.788 | 387 (68.1) | 123 (66.5) | 0.918 |
| Diastolic Blood Pressure | 237 (31.5) | 28 (43.1) | 0.702 | 172 (30.3) | 65 (35.1) | 0.534 |
| Heart rate | 119 (15.8) | 10 (15.4) | 0.999 | 90(15.9) | 29 (15.7) | 0.999 |
| Hemoglobin | 348 (46.2) | 30 (46.2) | 0.999 | 273 (48.1) | 75 (40.5) | 0.356 |
| Hematocrit (miss=26) | 390 (53.6) | 390 (49.2) | 0.995 | 298 (54.4) | 92(51.4) | 0.875 |
| Mean corpuscular volume | 221 (29.3) | 14 (21.5) | 0.785 | 161 (28.4) | 60 (32.4) | 0.666 |
| Mean corpuscular hemoglobin | 281 (37.3) | 20 (30.8) | 0.881 | 205 (36.1) | 76 (41.1) | 0.534 |
| Red Cell Distribution Width (miss=36) | 417 (58.2) | 43 (68.3) | 0.785 | 324 (59.8) | 93 (53.1) | 0.382 |
| White blood cells | 414 (55.0) | 29 (48.3) | 0.881 | 326 (57.4) | 88 (47.6) | 0.261 |
| Lymphocytes (miss=1) | 263 (35.0) | 24(36.9) | 0.995 | 196 (34.6) | 67 (36.2) | 0.918 |
| Platelets | 78 (10.4) | 9 (13.9) | 0.978 | 54 (9.5) | 24 (13.0) | 0.534 |
| Creatinine | 361 (47.9) | 30 (46.2) | 0.995 | 278 (48.9) | 83 (44.9) | 0.709 |
| Blood urea | 452 (60.0) | 43 (66.2) | 0.881 | 348 (61.3) | 104 (56.2) | 0.534 |
| Sodium | 115 (15.3) | 9 (13.9) | 0.995 | 85 (15.0) | 30 (16.2) | 0.918 |
| Potassium | 79 (10.5) | 9 (13.9) | 0.988 | 58 (10.2) | 21 (11.4) | 0.918 |
| Chloride (miss=26) | 208 (28.6) | 17 (28.2) | 0.995 | 156 (28.5) | 52 (29.1) | 0.999 |
| Protein, total (miss=6) | 620 (83.0) | 50 (78.2) | 0.881 | 477 (84.1) | 143 (79.4) | 0.457 |
| Albumin (miss=5) | 443 (59.2) | 40 (61.5) | 0.995 | 349 (61.8) | 94 (51.4) | 0.261 |
| Bilirubin Total (miss=27) | 78 (10.7) | 8 (12.3) | 0.995 | 60 (11.0) | 18 (10.1) | 0.999 |
| Phosphatase, alkaline | 131 (17.4) | 10 (15.4) | 0.995 | 97 (17.1) | 37 (20.0) | 0.747 |
| AST (miss=1) | 169 (22.5) | 15 (23.1) | 0.999 | 123 (21.7) | 46 (24.9) | 0.747 |
| ALT (miss=1) | 56 (7.7) | 2 (3.1) | 0.785 | 36 (6.6) | 20 (11.2) | 0.356 |
| Calcium (corrected) (miss=1) | 90 (12.0) | 11 (16.9) | 0.881 | 70 (12.4) | 20 (10.8) | 0.918 |
| Phosphorus, inorganic (miss=2) | 104 (13.8) | 12 (18.5) | 0.881 | 85 (15.0) | 19 (10.3) | 0.371 |
| TSH (miss=21) | 152 (20.8) | 14 (22.2) | 0.995 | 116 (20.9) | 36 (20.2) | 0.999 |
| Vitamin D (miss=97) | 313 (47.7) | 32 (54.2) | 0.881 | 238 (47.9) | 75 (47.2) | 0.999 |
| PTH (miss=116) | 268 (42.1) | 28 (51.9) | 0.785 | 214 (44.1) | 54 (35.3) | 0.356 |
| Vitamin B12 (miss=42) | 187 (26.3) | 17 (29.8) | 0.995 | 141 (26.2) | 46 (26.6) | 0.999 |
| Folic acid (miss=84) | 95 (14.2) | 9 (15.8) | 0.995 | 74 (14.8) | 21 (12.4) | 0.875 |
|  |  |  |  |  |  |  |
|  | | |  |  |  |  |

Miss- missing data; AST- aspartate aminotransferase ; ALT-alanine transaminase ; TSH-thyroid-stimulating hormone ; PTH- parathyroid hormone; Vitamin D- 25-hydroxy vitamin D;

P* -Difference between patients included and not included in the sample.

P**-Difference between patients included in the sample with hip fracture and pelvic fracture.

**Supplemental Table 3:** **Correlation between FI-Lab and the patients' socio-demographic and clinical characteristics: Hip and pelvic fracture patients**

|  | **Hip fracture (N=568)** | | | **Pelvic fracture (N=185)** | | |
| --- | --- | --- | --- | --- | --- | --- |
| **Variable** | **N** | **Spearman *r*** | **P** | **N** | **Spearman *r*** | **P** |
| Age, years | 568 | 0.109 | 0.126 | 185 | 0.036 | 0.626 |
| Body mass index | 562 | -0.069 | 0.339 | 179 | 0.155 | 0.089 |
| MMSE | 489 | -0.096 | 0.212 | 163 | -0.127 | 0.147 |
| CCI | 568 | 0.248 | 0.056 | 185 | 0.335 | 0.007 |
| LoS (days) | 568 | 0.051 | 0.339 | 185 | 0.174 | 0.063 |
| MRFS-R | 568 | -0.048 | 0.339 | 185 | -0.130 | 0.136 |
| Delta FIM | 568 | -0.037 | 0.380 | 185 | -0.113 | 0.147 |

CCI- the Charlson Comorbidity Index; MMSE-the Mini-Mental State Examination; LoS- length of stay in the geriatric department; MRFS-R - the Montebello Rehabilitation Factor Score-Revised; FIM-- the Functional Independence Measure.

**Supplemental Table 4:** **The association between dichotomous variables and FI-Lab groups: Hip and pelvic fracture patients**

| **Variables** | **Frailty status group by FI-Lab score-HIP fracture (N=568)** | | | | | | | | | **Frailty status group by FI-Lab score-Pelvic fracture (N=185)** | | | | | | | | |
| --- | --- | --- | --- | --- | --- | --- | --- | --- | --- | --- | --- | --- | --- | --- | --- | --- | --- | --- |
|  | **Robust (N=127)** | | **Mildly pre-frail (N=138)** | | **Moderately pre-frail (N=146)** | | **Frail (N=157)** | | **P (for trend)** | **Robust (N=54)** | | **Mildly pre-frail (N=50)** | | **Moderately pre-frail (N=39)** | | **Frail (N=42)** | | **P (for trend)** |
|  | **N** | **%** | **N** | **%** | **N** | **%** | **N** | **%** |  | **N** | **%** | **N** | **%** | **N** | **%** | **N** | **%** |  |
| **Socio-demographic** |  |  |  |  |  |  |  |  |  |  |  |  |  |  |  |  |  |  |
| Gender (female) [n (%)] | 101 | 79.5 | 100 | 72.5 | 97 | 66.4 | 89 | 56.7 | <0.001 | 46 | 85.2 | 36 | 72.0 | 29 | 74.4 | 31 | 73.8 | 0.220 |
| Family status (married) [n (%)] | 54 | 42.5 | 67 | 48.6 | 59 | 40.4 | 69 | 43.9 | 0.821 | 21 | 38.9 | 18 | 36.0 | 11 | 28.2 | 18 | 42.9 | 0.931 |
| Nursing caregiver (yes) [n (%)] (miss=43) | 71 | 55.9 | 79 | 57.2 | 80 | 54.8 | 98 | 62.4 | 0.289 | 28 | 51.9 | 25 | 50.0 | 26 | 66.7 | 31 | 73.8 | 0.009 |
| **Medical status** |  |  |  |  |  |  |  |  |  |  |  |  |  |  |  |  |  |  |
| Depression [n (%)] | 14 | 11.0 | 9 | 6.5 | 10 | 6.8 | 14 | 8.9 | 0.628 | 5 | 9.3 | 5 | 10.0 | 12 | 30.8 | 2 | 4.8 | 0.692 |
| Delirium [n (%)] | 23 | 18.1 | 23 | 16.7 | 27 | 18.5 | 30 | 19.1 | 0.724 | 2 | 3.7 | 6 | 12.0 | 3 | 7.7 | 9 | 21.4 | 0.016 |
| Any complication [n (%)] | 63 | 49.6 | 82 | 59.4 | 86 | 58.9 | 102 | 65.0 | 0.075 | 13 | 24.1 | 22 | 44.0 | 21 | 53.8 | 21 | 50.0 | 0.005 |
| **Functional status** |  |  |  |  |  |  |  |  |  |  |  |  |  |  |  |  |  |  |
| Patients with MRFS-R ≥50 [n (%)] | 90 | 70.9 | 100 | 72.5 | 100 | 68.5 | 102 | 65.0 | 0.198 | 43 | 79.6 | 36 | 72.0 | 24 | 61.5 | 28 | 66.7 | 0.090 |
| Discharged to LTC [n (%)] | 8 | 6.3 | 10 | 7.2 | 2 | 1.4 | 6 | 3.8 | 0.136 | 2 | 3.7 | 3 | 6.0 | 2 | 5.1 | 1 | 2.4 | 0.868 |

MRFS-R- the Montebello Rehabilitation Factor Score- Revised; LTC - long-term care; mis=missing

**Supplemental Table 5:** **All-cause mortality during the follow-up period: Hip and pelvic fracture patients**

| **Variables** | **HIP fracture (N=568)** | | | **Pelvic fracture (N=185)** | | |
| --- | --- | --- | --- | --- | --- | --- |
|  | **Alive (N=355)** | **Dead (N=213)** | **P** | **Alive (N=127)** | **Dead (N=58)** | **P** |
| Age (years) (Mean±SD) | 81.0±7.7 | 83.8±7.5 | <0.001 | 80.4±6.9 | 83.7±7.9 | 0.005 |
| Gender (female) [n (%)] | 259 (72.9) | 128 9 (60.1) | 0.002 | 104 (81.9) | 38 (65.5) | 0.023 |
| Depression (yes) [n (%)] | 32 (9.0) | 15 (7.0) | 0.436 | 18 (14.2) | 6 (10.3) | 0.638 |
| BMI, (Mean±SD) (miss=12) | 25.3±4.6 | 24.8±4.3 | 0.268 | 24.9±4.2 | 26.4±5.1 | 0.049 |
| MMSE, (Mean±SD) | 23.1±5.4 | 22.7±5.3 | 0.550 | 24.1±4.6 | 23.0±5.5 | 0.209 |
| Any complication (yes) [n (%)] | 197 (55.5) | 136 (62.9) | 0.053 | 50 (39.4) | 27 (46.5) | 0.422 |
| CCI TS (Mean±SD) | 3.8±2.7 | 4.7±2.9 | <0.001 | 3.5±2.8 | 5.0±3.5 | 0.003 |
| MRFS-R (Mean±SD) (Median) | 59.2±62.2 | 43.4±72.9 | 0.006 | 52.4±126.5 | 58.1±63.1 | 0.748 |
| FI-Lab (Mean±SD) | 0.32±0.10 | 0.37±0.11 | <0.001 | 0.30±0.10 | 0.38±0.12 | <0.001 |

CCI- the Charlson Comorbidity Index; MMSE-the Mini-Mental State Examination ; BMI- body mass index; MRFS-R- the Montebello Rehabilitation Factor Score - Revised

**Supplemental Table 6:** **Cox proportional hazard models for all-cause mortality during the first year: Hip fracture patients**

| **Model** | **Variables** | **Hip fracture (N=568)** | | | | **Pelvic fracture (N=185)** | | | |
| --- | --- | --- | --- | --- | --- | --- | --- | --- | --- |
|  |  | **Hazard Ratio** | **95.0% CI** | | **P** | **Hazard Ratio** | **95.0% CI** | | **P** |
|  |  |  | **Lower** | **Upper** |  |  | **Lower** | **Upper** |  |
| 1 | **FI-Lab score** |  |  |  |  |  |  |  |  |
|  | Robust | 1.000 |  |  |  |  |  |  |  |
|  | Mildly frail | 1.363 | 0.562 | 3.306 | 0.493 |  |  |  |  |
|  | Moderately frail | 1.558 | 0.667 | 3.638 | 0.306 |  |  |  |  |
|  | Severely frail | 2.748 | 1.235 | 6.115 | 0.013 |  |  |  |  |
|  | **Gender (Female)** | 0.558 | 0.347 | 0.898 | 0.016 |  |  |  |  |
|  | **Age (Years)** | 1.017 | 0.987 | 1.049 | 0.270 |  |  |  |  |
|  | **Any complication (yes)** | 1.961 | 1.144 | 3.359 | 0.014 |  |  |  |  |
|  | **CCI score** | 1.031 | 0.951 | 1.118 | 0.462 |  |  |  |  |
| 2 | **FI-Lab (continuous)** | 1.031 | 1.009 | 1.053 | 0.006 | 1.061 | 1.019 | 1.104 | 0.004 |
|  | **Gender (Male)** | 0.545 | 0.339 | 0.877 | 0.012 | 1.272 | 0.447 | 3.62 | 0.652 |
|  | **Age (Years)** | 1.018 | 0.988 | 1.049 | 0.242 | 1.101 | 1.023 | 1.185 | 0.01 |
|  | **Any complication (yes)** | 1.944 | 1.133 | 3.335 | 0.016 | 2.091 | 0.759 | 5.764 | 0.154 |
|  | **CCI score** | 1.029 | 0.949 | 1.117 | 0.489 | 1.045 | 0.904 | 1.208 | 0.551 |

CCI- the Charlson Comorbidity Index

Model 1 for Pelvic fracture is not presented due to instability in the estimates resulting from the very small number of deaths in the pelvic fracture subgroup (n=19). The limited number of events led to extreme hazard ratio values and implausibly wide confidence intervals, rendering the model unsuitable for meaningful interpretation.

**Supplemental Table 7:** **Cox proportional hazard models for all-cause mortality during entire follow-up period: Hip and pelvic fracture patients**

| **Model** | **Variables** | **HIP fracture (N=568)** | | | | **Pelvic fracture (N=185)** | | | |  |
| --- | --- | --- | --- | --- | --- | --- | --- | --- | --- | --- |
|  |  |  |  |  |  |  |  |  |  |  |
|  |  | **Hazard Ratio** | **95.0% CI** | | **P** | **Hazard Ratio** | **95.0% CI** | | **P** |  |
|  |  |  | **Lower** | **Upper** |  |  | **Lower** | **Upper** |  |  |
| 1 | **FI-Lab score** |  |  |  |  |  |  |  |  |  |
|  | Robust | 1.000 |  |  |  | 1.000 |  |  |  |  |
|  | Mildly frail | 1.058 | 0.672 | 1.668 | 0.807 | 1.523 | 0.626 | 3.707 | 0.354 |  |
|  | Moderately frail | 1.364 | 0.896 | 2.074 | 0.148 | 2.014 | 0.800 | 5.070 | 0.137 |  |
|  | Severely frail | 1.933 | 1.283 | 2.913 | 0.002 | 4.14 | 1.779 | 9.632 | <.001 |  |
|  | **Gender (Female)** | 0.77 | 0.579 | 1.025 | 0.073 | 0.599 | 0.333 | 1.075 | 0.086 |  |
|  | **Age (Years)** | 1.035 | 1.017 | 1.054 | <.001 | 1.083 | 1.039 | 1.128 | <.001 |  |
|  | **Any complication (yes)** | 1.557 | 1.171 | 2.071 | 0.002 | 1.561 | 0.905 | 2.692 | 0.109 |  |
|  | **CCI score** | 1.099 | 1.046 | 1.154 | <.001 | 1.091 | 1.006 | 1.183 | 0.036 |  |
| 2 | **FI-Lab (continuous)** | 1.023 | 1.01 | 1.036 | <.001 | 1.047 | 1.023 | 1.072 | <.001 |  |
|  | **Gender (Male)** | 0.763 | 0.574 | 1.014 | 0.062 | 0.633 | 0.356 | 1.124 | 0.119 |  |
|  | **Age (Years)** | 1.036 | 1.017 | 1.054 | <.001 | 1.085 | 1.041 | 1.131 | <.001 |  |
|  | **Any complication (yes)** | 1.556 | 1.17 | 2.071 | 0.002 | 1.504 | 0.867 | 2.607 | 0.147 |  |
|  | **CCI score** | 1.097 | 1.044 | 1.153 | <.001 | 1.087 | 1.000 | 1.182 | 0.05 |  |

CCI- the Charlson Comorbidity Index
